# Involvement of the activating receptor NKG2D in cutaneous hypersensitivity drug reactions

**DOI:** 10.1186/2045-7022-4-S3-P47

**Published:** 2014-07-18

**Authors:** Teresa Bellón, Marcial García, Elena Pardo, Salvador Escamochero, Ana María Fiandor, Pedro Herranz, Rosario Cabañas, Carlos González-Herrada

**Affiliations:** 1 Hospital La Paz Health Research Institute- IdiPAZ, Spain; 2 Hospital La Paz Health Research Institute- IdiPAZ, Research Unit, Spain; 3 Hospital Universitario La Paz, Allergy, Spain; 4 Hospital Universitario La Paz, Dermatology, Spain; 5 Hospital Universitario La Paz- IdiPAZ, Allergy, Spain; 6 Hospital Universitario de Getafe, Dermatology, Spain

## Background

Type IV drug hypersensitivity reactions with cutaneous manifestations (cADR) are highly variable in clinical appearance and degree of severity, ranging from maculopapular exanthema (MPE) to more severe disorders such as DRESS or SJS/TEN. Recent studies show NKp46+ cells in skin of drug-induced exanthemas, and NK cells expressing granulysin have been found in blister fluids from SJS/TEN patients, suggesting that NK cells can contribute to keratinocyte killing as well as CTLs. The balance of activating and inhibitory signals delivered by innate NK receptors determines the activity of NK cells. Among activating receptors, NKG2D is expressed in the cell membrane of NK cells and CTLs as a homodimer associated to the molecule DAP-10, which transduces the activating signal through the activation of PI-3 kinase. NKG2D ligands are HLA class I-like molecules MICA and MICB, and UL-16 binding proteins (ULBP). Their expression is upregulated in stress conditions.

## Objective

To explore the involvement of the activating receptor NKG2D in cADR.

## Methods

Quantitative RT- PCR was performed in skin biopsies from healthy donors, MPE, DRESS, and SJS/TEN patients to evaluate the expression of DAP-10, and NKG2D ligands (MICA, MICB and ULBP1-3). Degranulation assays were performed to investigate NKG2D-dependent activity in lymphocytes from blister fluids.

## Results

DAP-10 mRNA levels were found to be significantly increased in biopsies from DRESS patients compared to MPE and healthy skin. Nonetheless, the highest mRNA levels were detected in SJS/TEN biopsies. Among the five NKG2D ligands analyzed, only MICA and ULBP1 mRNA were identified in skin biopsies, and both were significantly upregulated in skin form SJS/TEN patients. Degranulation assays against NKG2D ligand-expressing cell lines revealed no NKG2D-driven cytolitic activity in CTLs and increased cytotoxicity by blister fluid NK cells compared to NKG2D ligand negative cells.

## Conclusion

DAP-10 mRNA levels suggest increased activity of NKG2D in lymphocytes infiltrating the skin in DRESS and SJS/TEN patients. However, the pattern of expression of NKG2D ligands indicates that NKG2D-dependen cytolytic activity might be more relevant in bullous diseases. Moreover, degranulation assays confirm NKG2D-dependen activity in blister fluid NK cells and suggest that NKG2D might be an activating receptor involved in keratinocyte killing by NK cells in SJS/TEN.

